# Litigation to Access Health Services: Ally or Enemy of Global Public Health?

**DOI:** 10.5334/aogh.2760

**Published:** 2020-02-07

**Authors:** Alexandre Martins, Sydney Allen

**Affiliations:** 1 Maquette University, US; 2 Medical College of Wisconsin, US

## Abstract

**Background:**

Some scholars and global health advocates argue that litigation is a strategy to advance public health care, especially in those countries that do not have specific legislation to guarantee access to basic health care services. However, strategic litigation has another side, known as judicialization of the right to health, particularly present in the Latin American region where most countries incorporate the right to health into their constitutions, but their citizens still struggle with health disparities.

**Objectives:**

Considering these two perspectives on litigation in health care, this paper examines the phenomenon of litigation in health care and its impact on public health in Brazil, where there is an ambiguous process of litigation in health care.

**Methods:**

Comparing the literature of both the use of strategic litigation for advancing public health and the judicialization of the right to health, this paper develops an ethical analysis of the impacts of strategic litigation for individuals and societies, using Brazil’s public health care system and its policies as case-study of the impact of court decisions on the management of the system.

**Findings:**

Supporters of strategic litigation present experiences in African countries using this strategy to access a specific medical service led to enforce the creation of health-related policies by authorities and policymakers. However, in Brazil, a country with the right to health guaranteed by its Constitution, strategic litigation creates access to health care for some individuals, but also results in complex sociomedical challenges with significant impact for public administration and distributive justice.

**Conclusions:**

Strategic litigation can lead to ambiguous results, which will depend on the local context and the existence or not of public health services and health-related policies. When this strategy is considered, ethical analysis helps to understand how litigation can both benefit and damage individuals’ health and the public health system in the complex context and diverse reality of Brazil. As a result, strategic litigation must be considered from an ethical perspective of prudence and discernment in a close interaction with the local reality, its particular circumstances, culture, policies, and laws.

## Introduction

In this paper, we will address the use of strategic litigation to access health care services from an ethical perspective, considering aspects of justice and distributive justice. Litigation has been a strategy that individuals and groups, including grassroots movements and holders of public offices in the judiciary and the legislative branches, have resorted to in an effort to achieve some form of health justice at both the individual and social level. This has increased the role of the courts in pressuring the executive body’s ability to manage the health system, especially in those countries with national public health systems and a web of policies to guarantee access to social services. Scholars argue that courts point out the political failures and executive mismanagement in upholding social rights, allowing a gap that will be filled by the judiciary branch.

Courts would act for the legal enforcement of health care in several ways, and the most common are: (1) individual enforcement in which courts rule to grant access to some benefits guaranteed by social rights to a single plaintiff; (2) negative injunction through which courts act to strike down benefits cuts or other laws that decrease or end social benefits; (3) structural enforcement through which courts act to guarantee a collective appeal from one or more cases that represent this collective request and thus lead to creation of policies with structural impact [1]. When one thinks of these three forms of legal enforcement in the context of accessing medical care and health-related policies, it is apparent that each one leads to different impacts on the health care system. However, it is difficult to measure the positive impacts because often enough, while a court decision has an immediate positive impact on the life of a single individual, the plaintiff, or on a particular social group, this generates a social-structural problem for the allocation of public resources that this court decision demands from the executive body. However, there are situations in which the court’s ruling forces the executive and legislative branches to create new policies and/or change strategies, priorities, and rethink public management. In this case, the result will be a structural shift to make a public good, such as health care, a right with new ways of accessibility.

The ambiguity of the impact of litigation on health care raises questions on the benefits of strategic litigation in global public health in terms of accessing justice by a single individual and distributive justice created by socioeconomic and political structures. Thereby, this is the central question of this paper: is strategic litigation an ally or an enemy of global public health? Our goal is not to submit a yes or no answer to this question. Perhaps this would only be possible in an abstract account, but it would require one to pick a side in their approach to justice, choosing between who or what takes priority: the need of a single individual or the maintenance of the social body prioritizing the collective. As we neither dismiss the health care needs of the individual nor the necessity of a sustainable public health system, we won’t suggest an abstract approach, but rather guide our account through concrete examples of strategic litigations and their consequences. Thus, we argue that litigation in health care must be considered in different ways depending on the reality and the local laws (or lack thereof) of each particular area. The reality of a country and its health-related laws would require different ways to address litigation as a positive or negative strategy for health care access by individuals and distributive justice.

### Litigation and Fairness

Supporters of litigation as a mechanism to advance access to health care stress that court decisions force the creation of laws and policies to guarantee access to medical services in countries that do not have specific legislation. Some also affirm that, even in countries that have health-related legislation and a public system, litigation forces to improve the system, to expand access, and to create institutions to improve public management. These two arguments must be placed in a context to make more sense because they are two different uses of litigation in distinct health contexts.

The first is the use of litigation in those countries with either no health-related policies and health system, or their policies and system are very limited, that is, many services related to health are not covered (this would be the case of a country with a public system that provides primary care, but it does not cover HIV medication, while its population has many cases of HIV infection). Considering this context, Tamar Ezer and Priti Patel argue that litigation is a strategy to advance public health care, especially in those countries that do not have specific legislation to guarantee their people access to basic health care services. Their argument relies on experiences in African countries that used “strategic litigation as an important tool to develop and enforce legal protections critical to health [2].” This strategy was important to join different social sectors, such as grassroots movements, media and health professionals, to influence policies, law, and practices that advance public health, with particular success in the context of HIV. Ezer and Patel narrow their definition of strategic litigation by using the perspective of litigation as “an intended impact beyond a particular case to influence broader change at the level of law, policy, practice, or social discourse [2].” This broad impact occurs when strategic litigation does not operate alone and isolated in the courts. Strategic litigation needs partners to raise the visibility of the case and thus create a public debate. With visibility, a litigation demonstrates that what an individual is requesting could be a right to a good that everyone should access, benefiting the development of the society. One example of this occurred in the context of HIV in South Africa. “The classic case is *Minister of Health v. Treatment Action Campaign*, where the Constitutional Court of South Africa required the government to provide medication to prevent mother-to-child transmission of HIV and reversed the government’s policy of denying necessary medication to pregnant HIV-positive women [2].” Similar processes of strategic litigation occurred in other African countries, such as Botswana, Kenya, and Malawi.

We also want to mention a positive impact of strategic litigation in Brazil, a country with an extreme increase of health litigation in recent years. Although Brazil institutionalized a health program for HIV in 1986, this program became a reality with free distribution of medications in 1996, after years of mobilizing the civil society and many lawsuits against the government for accessing HIV medication. This mobilization and strategic litigation were possible because the democratization of the country and the promulgation of the right to health as a constitutional mandate [3]. As a case study, Brazil shows that litigation has functioned to disturb the balance between public budgets and distributive justice in health. However, there are arguments suggesting that litigation in Brazil has also had positive impact. Danielle da Costa L. Borges, for example, argues: “Individual litigation for health care rights in Brazil has pushed forward policy changes that range from strengthening health technology and assessment processes to better health care governance through institutional dialogue between different state actors [3].” Borges demonstrates that the high number of litigations and court decisions in Brazil have forced public authorities from all bodies of government to work in collaboration among themselves and with public health experts. This led to the creation of institutions to help judges, prosecutor’s offices, and attorneys access technical advice regarding health needs and requests. Additionally, these institutions (e.g. CONITEC, NAT) also support public authorities and managers in the administration of the public health system and in the improvement of health services assessments [4]. This collaboration has shaped new forms of governance and decreased the number of litigations in the States that have used these institutions [5]. However, the concern raised here is that these new forms of collaboration and strategies have not improved access to health care services for those individuals who feel marginalized from the system; instead, they only created a kind of bureaucracy that prevents these individuals from bringing lawsuits against their local, state, and federal governments. Consequently, individuals do not access some sort of justice, and the necessity to improve distributive justice is ignored.

### Litigation and Distributive Justice: The Case of Brazil

To understand the phenomenon of litigation or judicialization of health care in Brazil, one needs to be aware of the Brazilian health system and the federal laws regulating it [6]. The Constitution of 1988, created after 25 year of dictatorship, states health as a right of all and the duty of the state is to guarantee that all people in Brazilian territory have access to the benefit that this right creates [7]. Following the Constitution, the *Leis Orgânicas de Saúde* 8.080/90 and 8.142/90 created the Unified Health System (SUS) based on the principles of universality, integrality, and equity, with social-participation, shared funding responsibility, and decentralized management [8, 9]. The universal and integral access to health care in Brazil opens a large avenue for the judicialization of the right to health when public authorities fail to fulfill their obligations. In practical terms, this means that someone who needs a medical service or a medication and is unable to obtain it in the public service would have to fight to access the service through a court decision. The main reason that makes this lawsuit possible – for a person who cannot receive the medical care he/she needs and judges to rule in his/her favor – is the Article 196 of Federal Constitution.

According to the National Council of Justice (CNJ), in 2016 Brazil had more than 300 thousand lawsuits related to the right to health [10, 11]. In a study published by Ribeiro and Hartmann, the main characteristics of the judicialization of the right to health are: “Judicial claims are individual, not collective; Most cases request the provision of drugs through SUS; Claims have a success rate of 90%; [and] Favorable rulings are not based on independent medical assessments, but rather on prescriptions of the plaintiffs’ physicians [12].”

There is an enormous and disorderly growth of legal actions in the last few years in all levels of the Brazilian juridical system. For example, in the last 10 years, only the Supreme Court had more than 3,800 lawsuits to judge [12]. This caused scholars from diverse fields, especially scholars from public health, public policies, and law, to study this phenomenon. The literature shows that the precarious situation of the SUS, which creates problems for people to access healthcare services, is the main cause leading patients and families to bring lawsuits to the courts [11,13]. It seems obvious that if a sick person needs medical services and the public system cannot meet his/her need, this person will bring a lawsuit to the court to have his/her right to health care guaranteed. Another reason for these lawsuits is to access a kind of health care that the SUS does not provide. This is specifically prevalent in the case of accessing medications. The SUS has a long list of medications that are provided to the population as part of the citizens’ right to health. However, in some cases someone needs a medication that is not provided by the SUS, because it is not yet available in Brazil or because it is very expensive. Studies show that most lawsuits concern a need to obtaining medications, with the majority requesting medications of high cost, including those that are not part of governmental drug formularies [14, 15].

The case of Brazil shows that strategic litigation creates an ethical dilemma between the need of an individual and distributive justice. On the one hand, a lawsuit can guarantee that a person has what he/she needs, and it is his/her right, a constitutional right. On the other hand, scholars who are dedicated to this issue in Brazil seem to agree that the judicialization of the right to health creates an extra-expenditure for public administrations that was not part of their budget. As a result, judicialization has a tremendous impact on the distribution of resources in society [12,15]. Biehl and Petryna even argue that “although lawsuits secure access for thousands of people, at least temporarily, this judicialization of the right to health generates intensely complex sociomedical realities and significant administrative and fiscal challenges which, officials argue, have the potential to widen inequalities in health care delivery [16].”

Nevertheless, when the political, legislative, and executive powers cannot create policies and implement actions and strategies to fulfill their obligation to guarantee healthcare services for the population, the judiciary power is activated to create access to a fundamental right protected by the Constitution. According to Costa, Motto, and Araújo, the judicialization to the right to health “justifies itself because, in the constitutional and infra-constitutional dimensions of this right in a context of the precarious reality of the Brazilian health, the Judiciary has the responsibility of defining guidelines for offering this public service [14].” And they add: “the Judiciary assumed the role of guaranteeing the exercise, implementation, and concretization of individual and fundamental rights, explicitly stated in the Constitution, when the executive and legislative powers are negligent [14].”

The previous and current administrations have issued policies favoring the private health sector [17]. These neoliberal policies created a process of dismantling the SUS and all its health policies [18] that aimed to guarantee the right to health established by the Brazilian Constitution. With a non-functioning public healthcare system, access to medical services tends to decrease, creating many obstacles for people to access services they need and marginalizing many others from their rights. This situation forces people to use the courts in order to have their rights honored. Consequently, the problem of judicialization of the right to health tends to multiply its cases every day. While health policies do not shift to a direction that promotes the expansion and strengthening of the SUS, courts will fulfill the gap left by the negligence of the legislative and executive powers, creating more challenges for public managers, people in need of healthcare assistance, and the society as a whole, especially for those who are poor and marginalized.

## Conclusion

As we stated at the beginning of this essay, we did not intend to provide a yes or no answer for the question “is strategic litigation an ally or enemy for global public health?” We endeavored to demonstrate that there are situations in which litigation in health care generates a positive social impact by favoring the creation of health-related policies that promote access to health care and even structural changes. As a result, distributive justice advances. However, there are other cases in which litigation made justice possible for an individual, but disturbed the system, backsliding distributive justice. More studies are needed to provide substantial data on the impact of litigation on people’s lives and health care systems. However, we argue that litigation in health care has two faces: one can be positive for single individuals and negative for the sustainability of the system, and another can be positive for both by promoting the development of the health care system and more accessibility to individuals. Therefore, strategic litigation must be considered from a perspective of prudence and discernment in close interaction with the local reality, its particular circumstances, culture, policies, and laws.
